# Genome-wide characterization and stress-responsive expression analysis of the cinnamoyl-CoA reductase gene family in soybean

**DOI:** 10.3389/fpls.2025.1657111

**Published:** 2025-09-01

**Authors:** Xin Li, Yunlong Li, Sinan Li, Minghao Sun, Quan Cai, Yan Sun, Shujun Li, Yue Yin, Tao Yu, Jianguo Zhang

**Affiliations:** Maize Research Institute, Heilongjiang Academy of Agricultural Sciences, Harbin, China

**Keywords:** soybean, cinnamoyl-CoA reductase, lignin biosynthesis, abiotic stress, gene expression, phylogenetic analysis

## Abstract

**Background:**

Cinnamoyl-CoA reductase (CCR) catalyzes the first step in lignin biosynthesis and is crucial for plant development and stress response. Although CCR genes are characterized in many plants, a complete analysis of the soybean CCR family and its response to abiotic stress is limited.

**Methods:**

We identified soybean CCR genes genome-wide using bioinformatics. Phylogenetics, gene structures, motifs, chromosomal distribution, and synteny were analyzed. Promoter regions were checked for cis elements. Expression patterns were studied across tissues and under four abiotic stresses (salt, alkaline, drought, and osmotic) using transcriptome data.

**Results:**

Fifteen CCR genes (*GmCCR1-GmCCR15*) were identified in the soybean genome, distributed across 12 chromosomes. Phylogenetic analysis revealed two major subfamilies with distinct evolutionary origins. The genes encode proteins ranging from 269 to 363 amino acids, with predicted subcellular localization mainly in the Golgi apparatus. Motif analysis identified 10 conserved domains, showing subfamily-specific distribution patterns. Promoter analysis uncovered abundant hormone-responsive and stress-related cis-elements, including abscisic acid response elements (*ABRE*), methyl jasmonate-responsive elements, and drought-responsive elements. Transcriptome analysis demonstrated tissue-specific expression patterns, with higher levels in roots, stems, and developing seeds. Under abiotic stress conditions, five genes (*GmCCR1*, *GmCCR4*, *GmCCR7*, *GmCCR8*, and *GmCCR15)* were significantly upregulated, while three genes (*GmCCR2*, *GmCCR11*, and *GmCCR13*) were downregulated or showed no response. Notably, *GmCCR4* exhibited the most dramatic changes in expression across all stress treatments, with peak upregulation occurring 3 hours post-treatment.

**Conclusions:**

This analysis explores soybean CCR gene evolution, structure, and divergence. Identifying stress-responsive CCR genes, especially *GmCCR4*, highlights a target for improving soybean stress tolerance via molecular breeding or genetic engineering. These findings enhance understanding of lignin regulation under stress and support the development of climate-resilient soybeans.

## 1 Introduction

Soybean is one of the world’s most important legume crops, providing essential protein and oil for human consumption and animal feed (Lamlom et al., 2020; Modgil et al., 2020). Global soybean production faces increasing challenges from abiotic stresses, including drought, salinity, and extreme temperatures, which can reduce yields by up to 50% (Islam et al., 2019). Climate change is expected to exacerbate these stress conditions, making the development of stress-tolerant cultivars a critical priority for sustainable agriculture (Do et al., 2019; Shahzad et al., 2021).

Plant responses to abiotic stress involve complex molecular mechanisms, including changes in cell wall composition through altered lignin biosynthesis (Nizam et al., 2024). Lignin, a complex phenolic polymer, offers structural support, facilitates water conductance, and provides defense against biotic and abiotic stresses (Pb et al., 2023). The phenylpropanoid pathway, which produces lignin precursors, is highly responsive to environmental stresses and plays a crucial role in a plant’s adaptation (Li et al., 2024).

Cinnamoyl-CoA reductase (CCR; EC 1.2.1.44) catalyzes the initial committed step in the monolignol branch of the phenylpropanoid pathway, converting hydroxycinnamoyl-CoA thioesters into their corresponding aldehydes (Huang et al., 2024). This enzyme is essential for the biosynthesis of the three main monolignols: p-coumaryl alcohol, coniferyl alcohol, and sinapyl alcohol, which serve as building blocks for lignin polymerization (Muro-Villanueva et al., 2022; Yin et al., 2022). Beyond its role in lignin biosynthesis, CCR participates in the production of defense-related compounds and contributes to plant stress tolerance (Ma, 2024). CCR genes have been characterized in various plant species, revealing diverse expression patterns and functional specialization. In Arabidopsis thaliana, two CCR genes (*AtCCR1* and *AtCCR2*) show distinct expression profiles, with *AtCCR1* primarily involved in developmental lignification and *AtCCR2* responding to stress and pathogen attack (Liu et al., 2021). Soybean contains multiple CCR genes with tissue-specific expression and differential responses to abiotic stress (Zheng et al., 2023). Similarly, maize (*Zea mays*) and wheat (*Triticum aestivum*) CCR genes exhibit functional diversification related to development and stress response (Liu, 2012).

Despite the agricultural significance of soybean and the vital function of CCR in stress tolerance, a comprehensive analysis of the soybean CCR gene family remains lacking. Previous studies have identified individual CCR genes in soybeans and demonstrated their involvement in stress responses (So et al., 2010; Aoyagi et al., 2014); however, a systematic characterization of the entire gene family is lacking. Understanding the evolutionary relationships, structural features, and expression patterns of soybean CCR genes is essential for elucidating their functional roles and identifying candidates for crop improvement. Recent advances in genomics and transcriptomics have provided powerful tools for the comprehensive analysis of gene families. The availability of high-quality soybean genome sequences enables the accurate identification of genes and structural analysis (Cannon and Shoemaker, 2012). Transcriptome sequencing enables the detailed characterization of gene expression patterns across various tissues and stress conditions (Severin et al., 2010). These approaches, combined with comparative genomics and phylogenetic analysis, can provide valuable insights into the evolution of gene families and their functional divergence.

Salt and alkaline stress represent significant constraints for soybean production, particularly in regions with saline soils (Ren et al., 2024). China, despite being the center of origin for soybean, has become the world’s largest importer due to limited arable land and increasing domestic demand (Qiu et al., 2013). Approximately 36.9 million hectares of Chinese agricultural land are affected by salinity and alkalinity, limiting soybean cultivation in these areas (Ren et al., 2024). Developing salt-tolerant soybean varieties could significantly increase domestic production and reduce import dependence.

The phenylpropanoid pathway, including CCR-mediated lignin biosynthesis, is known to be responsive to salt stress in various plant species (Neves et al., 2010). Salt stress can alter lignin content and composition, affecting cell wall properties and plant tolerance mechanisms (Chun et al., 2019).

In this study, we conducted a comprehensive genome-wide analysis of the soybean CCR gene family, including phylogenetic relationships, gene structures, conserved motifs, chromosomal distribution, and synteny analysis. We examined promoter regions for stress-responsive cis-regulatory elements and analyzed expression patterns across different tissues and under multiple abiotic stress conditions. Our objectives were to: (1) identify and characterize all CCR genes in the soybean genome; (2) investigate their evolutionary relationships and structural features; (3) analyze their expression patterns in different tissues and developmental stages; (4) evaluate their responses to abiotic stress conditions; and (5) identify candidate genes for improving soybean stress tolerance. This comprehensive analysis provides new insights into the evolution and functional diversification of soybean CCR genes and identifies promising targets for developing stress-tolerant soybean varieties through molecular breeding or genetic engineering approaches.

## 2 Materials and methods

### 2.1 Genome-wide identification of CCR gene family members

Soybean CCR gene family members were identified through comprehensive database searches using BLAST algorithms on NCBI (http://www.ncbi.nlm.nih.gov) and Phytozome v13 (https://phytozome.jgi.doe.gov/pz/portal.html). Candidate genes were screened based on the presence of conserved domains characteristic of cinnamoyl-CoA reductase (EC 1.2.1.44) using SMART domain analysis (http://smart.embl-heidelberg.de). Genes containing the conserved P-kinase domains (PF01370; PF01073) and high amino acid sequence similarity to Arabidopsis thaliana CCR proteins were selected as potential soybean CCR family members. Physicochemical properties of identified CCR proteins, including molecular weight, isoelectric point, and instability index, were analyzed using ExPASy ProtParam (https://web.expasy.org/protparam/). Subcellular localization predictions were conducted using Cell-PLoc 2.0 (http://www.csbio.sjtu.edu.cn/bioinf/Cell-PLoc-2/).

### 2.2 Phylogenetic analysis and protein domain architecture

CCR protein sequences from A. thaliana, Oryza sativa, Zea mays, and Triticum aestivum were retrieved from Phytozome based on EC classification (1.2.1.44) and conserved domain analysis. Multiple sequence alignments were performed using MUSCLE, and phylogenetic relationships were inferred with the neighbor-joining method implemented in MEGA11 with 1,000 bootstrap replicates. Phylogenetic trees were visualized and annotated using iTOL (http://itol.embl.de). Protein domain architecture was analyzed using Phytozome annotations and visualized with IBS software to illustrate domain organization and conservation patterns across family members.

### 2.3 Motif composition and gene structure analysis

Conserved motifs in soybean CCR proteins were identified using MEME Suite (https://meme-suite.org/meme/tools/meme) with default parameters, limiting the analysis to 10 motifs. Gene structure analysis, including exon-intron organization, was conducted using genome annotation files downloaded from Phytozome v13. Both motif distribution and gene structure were visualized with TBtools software.

### 2.4 Promoter analysis and cis-regulatory elements

Promoter sequences (2,000 bp upstream of the translation start site) for each *GmCCR* gene were obtained from the Phytozome database. Cis-acting regulatory elements were predicted using PlantCARE (https://bioinformatics.psb.ugent.be/webtools/plantcare/html/), focusing on stress-responsive, hormone-responsive, and tissue-specific elements. Results were visualized with TBtools for comparative analysis among family members.

### 2.5 Synteny and collinearity analysis

Syntenic relationships of soybean CCR genes were examined both within the soybean genome (segmental duplications) and between soybean and other plant species (*A. thaliana, O. sativa, Z. mays*, and *T. aestivum*). Collinearity analysis was conducted using TBtools with default settings to identify orthologous and paralogous gene pairs and to visualize syntenic blocks.

### 2.6 Plant material and abiotic stress treatments

The soybean cultivar Dongnong 50 (DN50), developed in our laboratory, was selected for this study based on its specific responses to abiotic stress. Seeds were surface-sterilized with 75% ethanol for 30 seconds, followed by 2.5% sodium hypochlorite for 10 minutes, and rinsed three times with sterile distilled water. Seeds were germinated and grown in plastic pots (20×20 cm) in a fully controlled, climate-controlled glasshouse at the Soybean Research Institute of Heilongjiang Academy of Agriculture Science. Plants were cultivated in a controlled environment growth chamber (Model PGC-15, Conviron, Winnipeg, Canada) under the following standardized conditions: 16/8 h light/dark photoperiod, photosynthetic photon flux density (PPFD) of 300 μmol m^-^² s^-^¹ provided by LED panels (400 – 700 nm spectrum), day/night temperatures of 25 ± 2 °C/20 ± 2 °C, relative humidity maintained at 60 ± 5%, and CO_2_ concentration of 400 ± 50 ppm. Light intensity was measured using a quantum sensor (LI-190R, LI-COR, Lincoln, NE, USA) and maintained consistently throughout the growth period. Plants were grown until the first trifoliate leaf was fully expanded before stress treatments were applied. Four different abiotic stress treatments were applied to evaluate the expression responses of *GmCCR* genes at the first trifoliate leaf stage. Salt stress was imposed by treating plants with 120 mM NaCl solution prepared by dissolving sodium chloride in distilled water and applied to the growth medium. Alkaline stress was applied using 100 mM NaHCO_3_ solution to simulate the high pH and bicarbonate conditions commonly found in saline-alkaline soils prevalent in northeastern China. Drought stress was simulated using 20% polyethylene glycol 6000 (PEG-6000) solution, prepared by slowly dissolving the polymer in distilled water at room temperature with continuous stirring until completely dissolved. Osmotic stress was applied using 200 mM mannitol solution prepared by dissolving D-mannitol in distilled water to create controlled osmotic conditions. Control plants (0 h samples) received normal growth conditions without any stress agents and served as the baseline for comparison.

### 2.7 Sample collection and RNA extraction

Root tissues were harvested at 0, 1, 3, 6, 12, and 24 hours post-treatment to capture the temporal dynamics of stress responses, with the 0-hour untreated samples serving as controls for each experiment. For each time point and treatment combination, biological replicates were collected to ensure statistical robustness. Root samples were immediately frozen in liquid nitrogen upon collection and stored at -80 °C until RNA extraction to preserve RNA integrity and prevent degradation. Total RNA was extracted from root samples using TRIzol reagent (Invitrogen, Carlsbad, CA, USA) following the manufacturer’s protocol. The extraction procedure involved tissue homogenization in TRIzol reagent, phase separation with chloroform, RNA precipitation with isopropanol, and washing with 75% ethanol. RNA integrity was verified by 1% agarose gel electrophoresis to check for the presence of intact 28S and 18S ribosomal RNA bands. RNA concentration and purity were quantified using a NanoDrop spectrophotometer (Thermo Scientific, Waltham, MA, USA), with only samples showing A260/A280 ratios between 1.8 and 2.2 being used for downstream applications.

### 2.8 RNA-seq library construction and transcriptome analysis

RNA-seq libraries were constructed from high-quality RNA samples using standard protocols for Illumina sequencing. Library preparation included mRNA purification, fragmentation, cDNA synthesis, adapter ligation, and PCR amplification, with library quality and quantity assessed using appropriate quality control measures. RNA-seq libraries were constructed using the TruSeq RNA Sample Preparation Kit v2 (Illumina Inc., San Diego, CA, USA) following the manufacturer’s protocol. Libraries were sequenced on an Illumina NovaSeq 6000 platform (Illumina Inc., San Diego, CA, USA) using 2×150 bp paired-end sequencing chemistry at the Beijing Genomics Institute (BGI, Shenzhen, China). Sequencing depth averaged 30 million clean reads per sample to ensure adequate coverage for differential expression analysis. Raw sequencing data were processed through quality control pipelines to remove low-quality reads and adapter sequences. Raw RNA-seq reads were processed using standard bioinformatics pipelines, with quality control performed using FastQC, and reads trimmed and filtered as necessary. Clean reads were aligned to the soybean reference genome (Wm82.a4.v1) using appropriate alignment software. Gene expression levels were quantified and normalized as transcripts per million (TPM) to account for differences in sequencing depth and gene length. Differential expression analysis was performed to identify genes showing significant changes in expression between treated and control samples.

### 2.9 Expression pattern analysis and statistical analysis

Expression patterns of *GmCCR* genes were analyzed using the processed transcriptome data, with temporal expression profiles generated for each gene across the six time points (0, 1, 3, 6, 12, and 24 hours) under each stress treatment condition. The sequences of primers used in this study are listed in **Supplementary Table S1**. Genes showing significant differential expression were identified based on statistical criteria, including fold change thresholds and adjusted p-values. Statistical analyses were performed using GraphPad Prism 9.5 software, with expression data analyzed using two-way ANOVA with treatment and time as factors, followed by Tukey’s multiple comparison test for *post-hoc* analysis. Statistical significance was set at p < 0.05, and the experimental design included appropriate biological replicates for each treatment and time point combination to ensure statistical power and reliability of the results.

## 3 Results

### 3.1 Genome-wide identification and comprehensive characterization of soybean CCR genes

**Figure 1:**
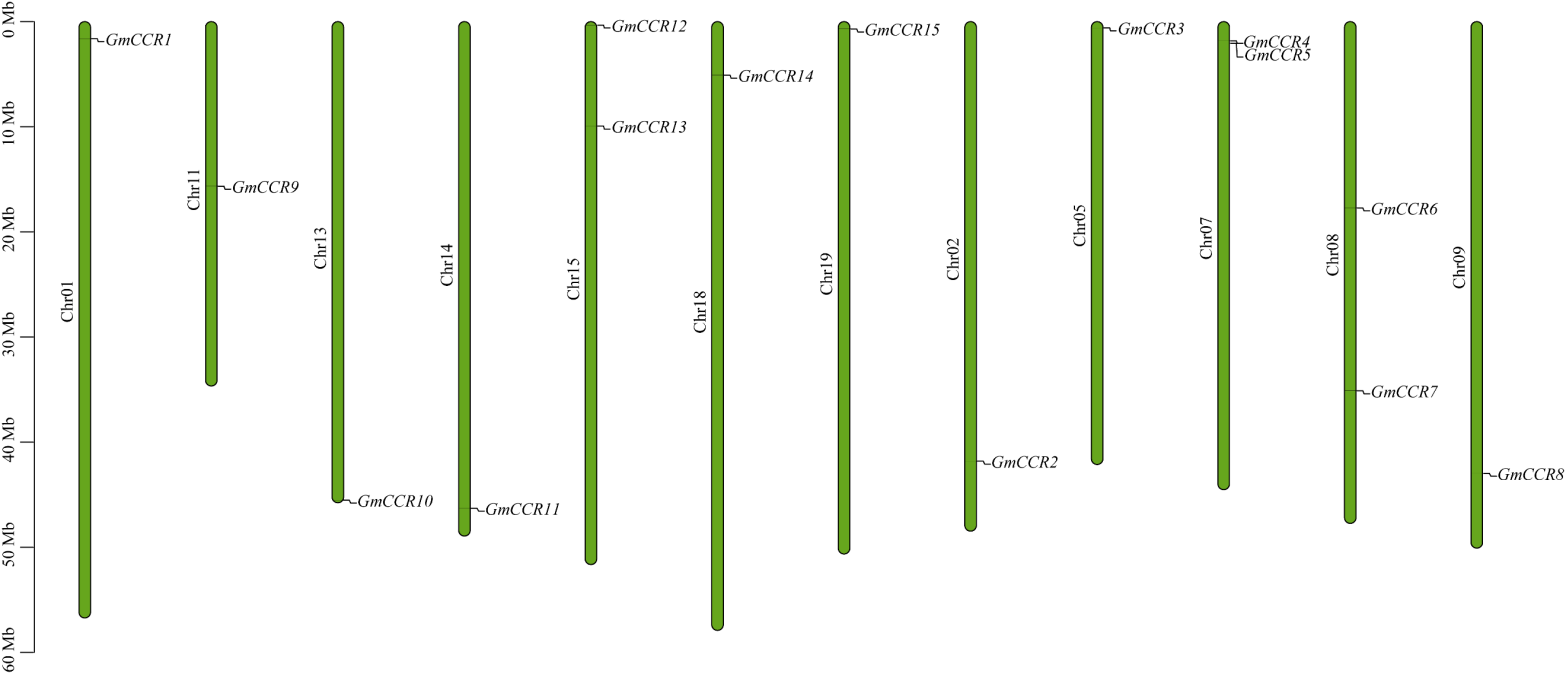
Chromosomal locations of CCR genes. Each vertical bar represents one chromosome. The chromosome number appears to the left of each chromosome. The locations of GmCCRs were mapped according to the soybean genome.

To systematically identify all members of the CCR gene family in soybean, we performed comprehensive BLAST searches against the NCBI and Phytozome v13 databases using known CCR protein sequences from *Arabidopsis thaliana* as queries. Following stringent filtering criteria based on conserved domain analysis and sequence similarity thresholds (>40% identity and E-value <1e-5), we identified 15 putative *GmCCR* genes distributed across 12 of the 20 soybean chromosomes (**Figure 1**, **Table 1**). The identified *GmCCR* genes were systematically named *GmCCR1* through *GmCCR15* based on their chromosomal positions and phylogenetic relationships. Chromosomal distribution analysis revealed that these genes are present on chromosomes 01, 02, 05, 07, 08, 09, 11, 13, 14, 15, 18, and 19, with chromosome 07, 08, and 15 each harboring two *GmCCR* genes, while the remaining chromosomes contain single genes. Notably, chromosomes 03, 04, 06, 10, 12, 16, 17, and 20 lack CCR genes, indicating non-random distribution patterns that may reflect evolutionary constraints or functional clustering. Detailed analysis of the coding sequences revealed substantial variation in gene length and encoded protein properties. The coding sequence lengths ranged from 807 bp (*GmCCR3*) to 1,089 bp (*GmCCR15*), corresponding to proteins of 269 – 363 amino acids. The predicted molecular weights varied from 29.84 kDa (*GmCCR3*) to 40.34 kDa (*GmCCR15*), while theoretical isoelectric points (pI) ranged from 5.24 (*GmCCR1*) to 6.94 (*GmCCR3*), indicating diverse biochemical properties that may reflect functional specialization. Instability index calculations revealed that 12 out of 15 GmCCR proteins (80%) were classified as stable (instability index <40), with only *GmCCR7*, *GmCCR10*, and *GmCCR14* showing instability indices above 40, suggesting potential regulatory roles or context-dependent stability. The grand average of hydropathicity (GRAVY) values were consistently negative (ranging from -0.142 to -0.387), indicating that all GmCCR proteins are hydrophilic, consistent with their predicted enzymatic functions in aqueous cellular environments.

**Table 1:** Characteristics of soybean CCR gene family members.

Gene Name	Gene ID	start	end	CDS (bp)	Amino Acids	MW (kDa)	pI	Subcellular Localization
*GmCCR1*	*Glyma.01G017100.Wm82.a4.v1*	1634012	1637492	900	300	33.77	5.24	Cytoplasm
*GmCCR2*	*Glyma.02G230500.Wm82.a4.v1*	41810217	41812913	963	321	35.22	6.07	Golgi apparatus
*GmCCR3*	*Glyma.05G006600.Wm82.a4.v1*	589984	593584	807	269	29.84	6.94	Cytoplasm
*GmCCR4*	*Glyma.07G023700.Wm82.a4.v1*	1825608	1828774	999	333	36.60	6.80	Golgi apparatus
*GmCCR5*	*Glyma.07G026300.Wm82.a4.v1*	2055071	2058068	966	322	35.79	6.41	Golgi apparatus
*GmCCR6*	*Glyma.08G218100.Wm82.a4.v1*	37557106	37562166	1002	334	36.80	5.95	Golgi apparatus
*GmCCR7*	*Glyma.08G270600.Wm82.a4.v1*	35110596	35122596	894	298	33.49	5.29	Chloroplast, Cytoplasm
*GmCCR8*	*Glyma.09G205700.Wm82.a4.v1*	42977627	42980853	900	300	33.79	5.33	Cytoplasm
*GmCCR9*	*Glyma.11G164700.Wm82.a4.v1*	15648078	15653438	966	322	34.96	5.65	Golgi apparatus
*GmCCR10*	*Glyma.13G369800.Wm82.a4.v1*	45526206	45528873	1017	339	37.13	6.56	Chloroplast, Golgi apparatus
*GmCCR11*	*Glyma.14G197600.Wm82.a4.v1*	46288253	46290941	1083	361	40.06	6.30	Golgi apparatus
*GmCCR12*	*Glyma.15G003600.Wm82.a4.v1*	332064	337381	1011	337	36.83	6.08	Golgi apparatus
*GmCCR13*	*Glyma.15G125100.Wm82.a4.v1*	9930652	9933697	993	331	35.70	5.41	Golgi apparatus
*GmCCR14*	*Glyma.18G057900.Wm82.a4.v1*	5092694	5097651	966	322	35.13	5.55	Golgi apparatus
*GmCCR15*	*Glyma.19G006900.Wm82.a4.v1*	686555	692466	1089	363	40.34	6.80	Chloroplast, Cytoplasm

Subcellular localization predictions using multiple algorithms (Cell-PLoc 2.0, TargetP, and ChloroP) revealed interesting distribution patterns. The majority of *GmCCR* proteins (9 out of 15, 60%) were predicted to localize to the Golgi apparatus, consistent with their role in lignin precursor synthesis and modification. Three proteins (*GmCCR1*, *GmCCR3*, and *GmCCR8*) were predicted to be cytoplasmic, while three others (*GmCCR7*, *GmCCR10*, and *GmCCR15*) showed dual localization potential, with predictions indicating possible targeting to both chloroplasts and cytoplasm or Golgi apparatus. This diverse subcellular distribution suggests functional compartmentalization within the CCR gene family, potentially allowing for tissue-specific or development-stage-specific regulation of lignin biosynthesis. The presence of chloroplast-targeted CCRs is particularly interesting, as it may indicate additional roles in specialized metabolic pathways beyond traditional lignin biosynthesis.

### 3.2 Phylogenetic relationships and evolutionary classification

**Figure 2:**
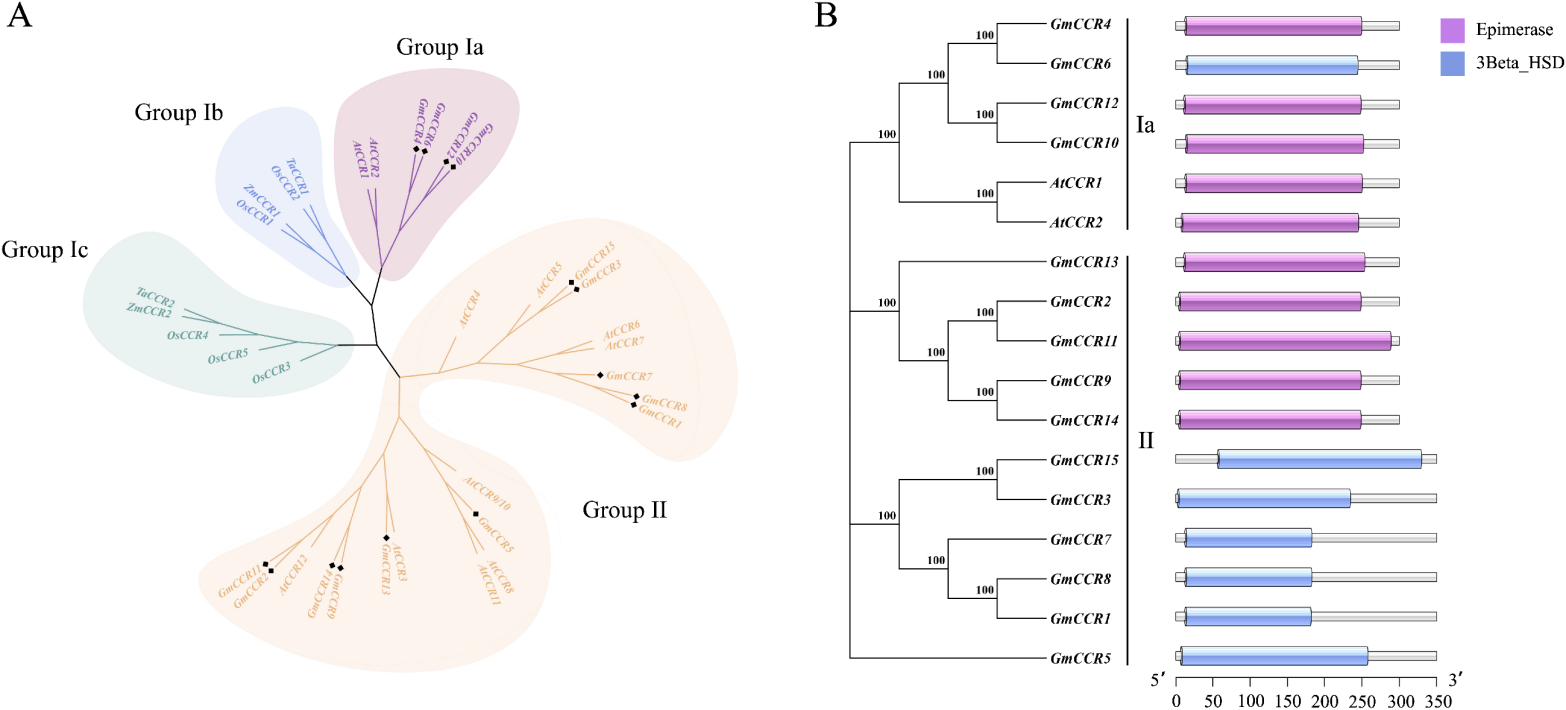
Phylogenetic relationships and domain architecture of soybean CCR genes. **(A)** Phylogenetic tree of steroidogenic enzyme gene families. Maximum likelihood phylogenetic tree showing evolutionary relationships among steroidogenic enzyme genes. Genes are clustered into four main groups: Group Ia, Group Ib, Group Ic, and Group II. Bootstrap values (>70) are indicated at major nodes. The scale bar represents evolutionary distance. **(B)** Comparative gene expression analysis of epimerase and 3β-hydroxysteroid dehydrogenase activities. A horizontal bar chart shows the relative expression levels of steroidogenic genes under two experimental conditions. Purple bars represent epimerase enzyme activity levels, while blue bars represent 3β-hydroxysteroid dehydrogenase (3Beta HSD) activity levels.

To understand the evolutionary relationships of soybean CCR genes, we constructed a phylogenetic tree using 35 CCR sequences from five plant species: 15 from soybean, 11 from *A. thaliana*, 5 from *O. sativa*, 2 from *Z. mays*, and 2 from *T. aestivum*. The phylogenetic analysis revealed four distinct subfamilies (Ia, Ib, Ic, and II) consistent with previous classifications (**Figure 2A**). Subfamily Ia contained both monocot and dicot sequences and is considered the “true CCR” group, with established roles in lignin biosynthesis. Notably, *GmCCR2*, *GmCCR4*, *GmCCR10*, and *GmCCR12* clustered with functionally characterized *AtCCR1* and *AtCCR2*, suggesting multifunctional hydroxycinnamoyl-CoA reductase activity. Subfamily Ib consisted exclusively of monocot sequences with proven lignin biosynthesis functions. Subfamily Ic contained monocot CCRs associated with plant defense responses. Subfamily II comprised 9 *AtCCR*-like and 12 *GmCCR*-like proteins requiring further functional characterization. Conserved domain analysis using the SMART database revealed consistent protein architecture across all soybean CCR family members (**Figure 2B**). All proteins contained the characteristic NAD(P)-binding domain (pfam01370) and the aldehyde dehydrogenase catalytic domain (pfam00171), essential for CCR enzymatic activity. Additionally conserved regions included substrate-binding domains and regulatory motifs that distinguish CCR proteins from other members of the short-chain dehydrogenase/reductase superfamily. The domain organization showed high conservation within subfamilies, with Subfamily Ia members displaying the most typical CCR architecture, while Subfamily II members exhibited some variations in domain boundaries and accessory motifs, consistent with their proposed functional diversification.

### 3.3 Conserved motif composition and gene structure organization

**Figure 3:**
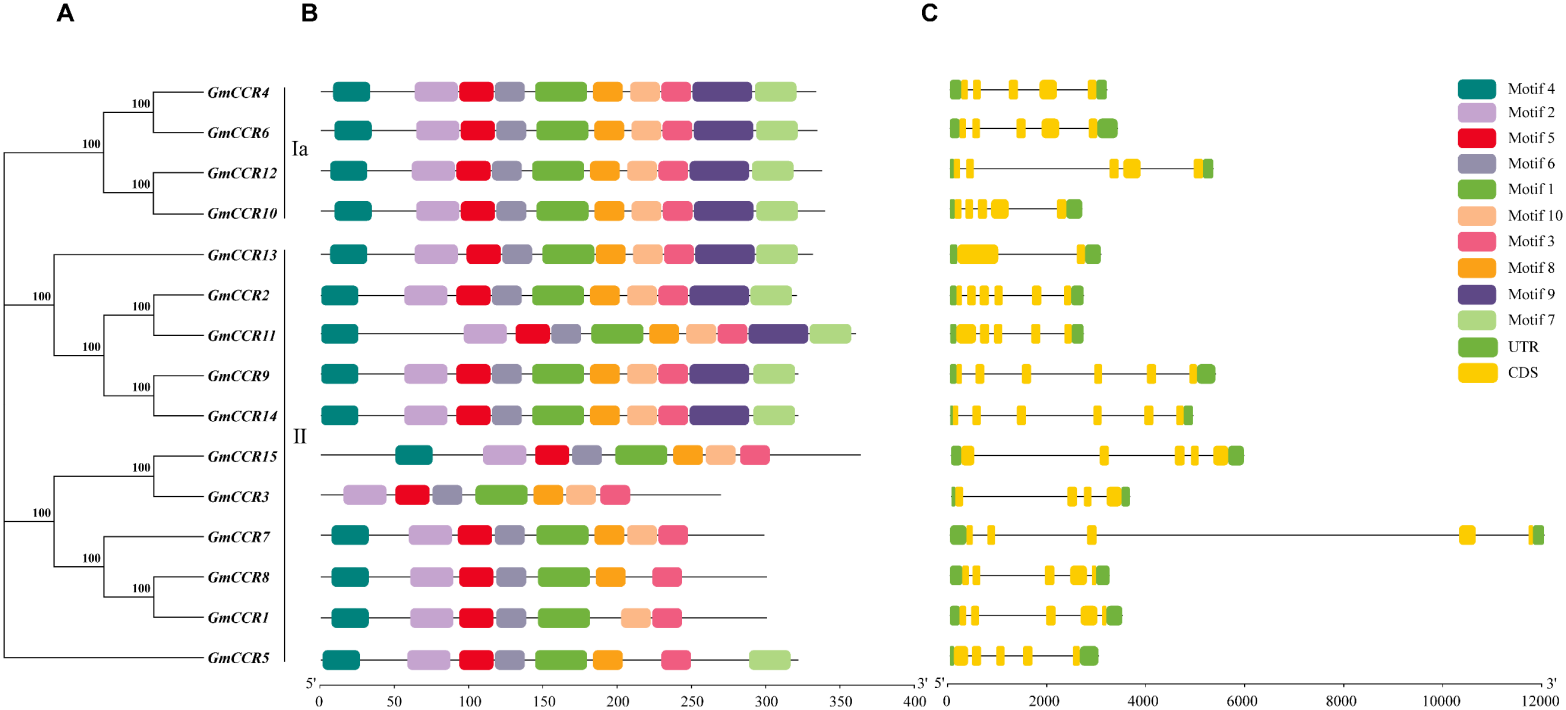
Conserved motif composition and gene structure analysis of soybean CCR genes. **(A)**, shows a maximum likelihood phylogenetic tree of GmCCR genes with bootstrap support values (>70) indicated at nodes. Genes are classified into two major groups (Ia and II) based on evolutionary relationships. Scale bar represents evolutionary distance (0.05 substitutions per site). **(B)** displays the conserved motif organization within each gene. Different colored boxes represent distinct conserved motifs identified through MEME analysis. **(C)** illustrates the exon-intron gene structure with exons depicted as yellow boxes connected by black lines indicating introns. The scale bars indicate sequence length in base pairs (bp), with the motif panel scaled 0 – 350 bp and the gene structure panel scaled 0-15,000 bp. This analysis reveals the evolutionary conservation and divergence patterns within the *GmCCR* gene family members.

Using the MEME Suite with optimized parameters (motif width 6 – 50 amino acids, maximum 10 motifs), we identified 10 highly conserved motifs across the 15 soybean CCR proteins (**Figures 3A, C**). The motif analysis revealed both conserved and subfamily-specific patterns that provide insights into functional evolution. Five core motifs (Motifs 1, 2, 3, 5, and 6) were present in all family members, representing essential structural elements for CCR function. Motif 1 (29 amino acids) contains the NAD(P)-binding signature sequence and is in the N-terminal region of all proteins. In comparison, Motif 2 (21 amino acids) represents part of the catalytic domain essential for substrate binding. Motif 3 (25 amino acids) contains conserved residues critical for cofactor specificity, Motif 5 (15 amino acids) forms part of the active site architecture, and Motif 6 (18 amino acids) is involved in protein stability and proper folding. Subfamily-specific motifs included Motifs 7 and 9, which were present in all Subfamily Ia members and 6 out of 11 Subfamily II members, potentially conferring enhanced catalytic efficiency. Motifs 4, 8, and 10 showed variable presence across family members, suggesting roles in functional specialization or regulatory interactions. The differential distribution of motifs 7 and 9 in Subfamily II members (*GmCCR5*, *GmCCR6*, *GmCCR13*, *GmCCR14*, and *GmCCR15* lack these motifs) provides molecular evidence for functional diversification within this expanded subfamily. Gene structure analysis revealed considerable variation in exon-intron organization among *GmCCR* genes, ranging from 1 to 4 exons per gene. Interestingly, the gene structure patterns closely correlated with phylogenetic relationships, with Subfamily Ia members consistently showing 2 – 3 exons with conserved intron positions, indicating structural constraint due to functional importance, while Subfamily II members displayed more variable structures (1 – 4 exons), suggesting relaxed selective pressure allowing structural diversification. Analysis of intron splicing phases revealed that 78% of introns were phase-0, consistent with the preservation of reading frames during exon shuffling events. The correlation between gene structure and phylogenetic classification suggests that structural evolution paralleled functional divergence, with more conserved structures in functionally constrained genes (Subfamily Ia) and increased structural flexibility in potentially neo functionalized genes (Subfamily II).

### 3.4 Comprehensive promoter analysis and regulatory element characterization

**Figure 4:**
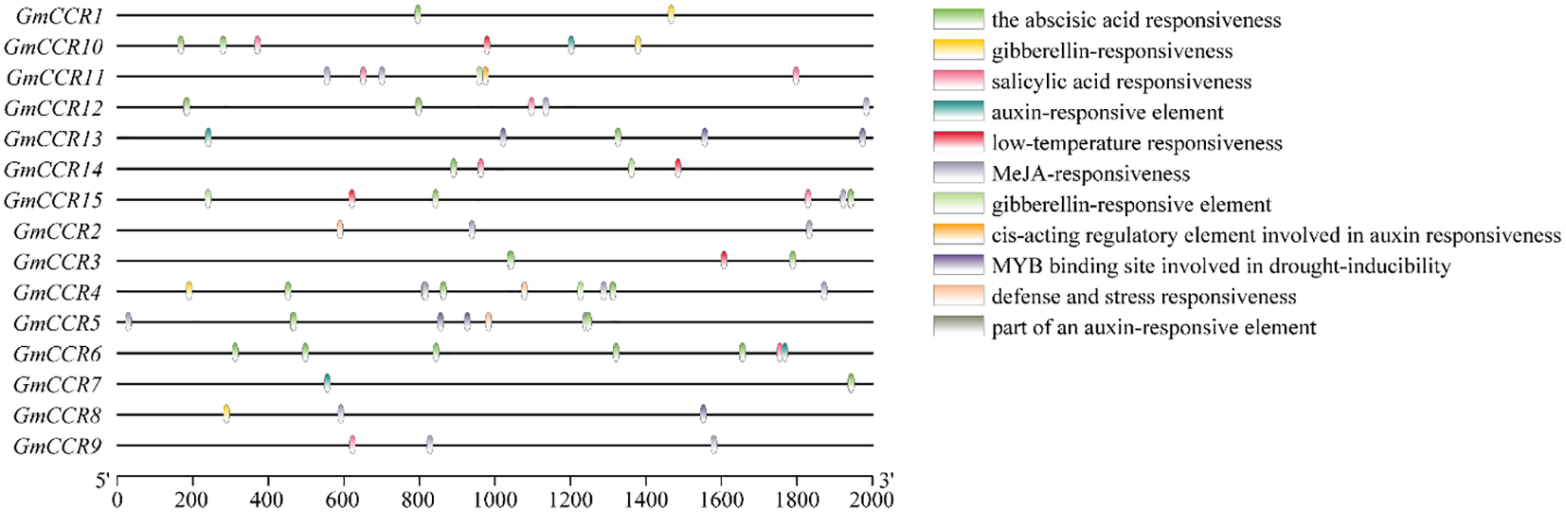
Cis-regulatory element analysis in soybean CCR gene promoters. Distribution and abundance of cis-acting regulatory elements identified in 2-kb upstream promoter sequences of *GmCCR* genes using PlantCARE database analysis. Functional groups categorize elements: hormone-responsive elements (ABA, abscisic acid; MeJA, methyl jasmonate; IAA, indole-3-acetic acid; GA, gibberellic acid; SA, salicylic acid), stress-responsive elements (TC-rich repeats for defense and stress response; LTR, low-temperature responsive), light-responsive elements, and tissue-specific elements. Genes are ordered according to phylogenetic subfamilies. The presence of multiple stress and hormone-responsive elements suggests complex transcriptional regulation of CCR genes in response to environmental stimuli.

Analysis of 2-kb upstream promoter regions using the PlantCARE database identified a total of 847 cis-regulatory elements across all 15 *GmCCR* promoters, with an average of 56.5 elements per promoter (**Figure 4**). The elements were categorized into several functional groups, with hormone-responsive elements comprising 324 total elements (38.3% of all elements). ABA-responsive elements (ABRE) were present in 14 out of 15 promoters with an average of 3.2 per promoter, indicating strong integration with drought and salt stress signaling. MeJA-responsive elements (TGACG-motif, CGTCA-motif) were found in 13 out of 15 promoters, suggesting roles in defense responses and secondary metabolism. In contrast, auxin-responsive elements (TGA-element, AuxRR-core) were present in 12 out of 15 promoters, potentially linking CCR expression to developmental processes. GA-responsive elements (P-box, GARE-motif) were identified in 10 out of 15 promoters, indicating involvement in growth regulation, and SA-responsive elements (TCA-element) were found in 8 out of 15 promoters, suggesting roles in pathogen defense. Stress-responsive elements totaled 198 elements (23.4% of all elements), with drought-responsive elements (MBS, DRE) present in all 15 promoters, with *GmCCR8* showing the highest density (8 elements). Low-temperature responsive elements (LTR) were found in 12 out of 15 promoters, indicating cold stress responsiveness. In comparison, heat shock elements (HSE) were present in 9 out of 15 promoters, suggesting thermotolerance roles, and TC-rich repeats were identified in 11 out of 15 promoters, associated with defense and stress responses. Comparative analysis revealed distinct regulatory patterns between subfamilies, with Subfamily Ia promoters enriched in developmental regulatory elements (CCGTCC-box, CAT-box) and showing higher densities of hormone-responsive elements, consistent with their roles in constitutive lignin biosynthesis. In contrast, Subfamily II promoters showed greater diversity in stress-responsive elements and tissue-specific regulatory motifs, supporting their proposed roles in specialized or inducible functions.

### 3.5 Synteny analysis reveals gene duplication patterns

**Figure 5:**
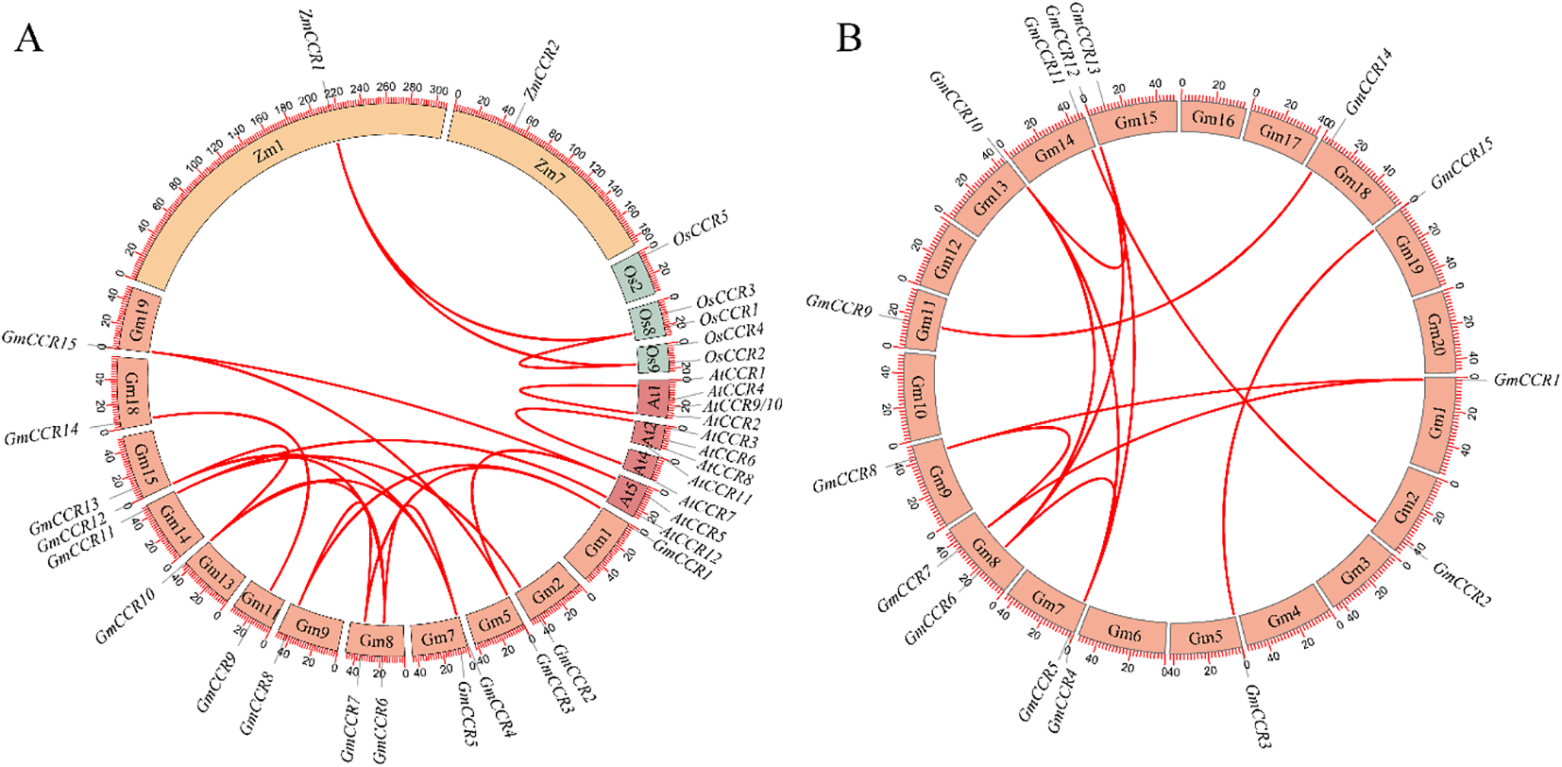
Synteny and collinearity analysis of CCR genes. **(A)** Interspecies synteny analysis of CCR gene families across four plant species. Chromosome numbers are indicated around the circle. A total of 20 syntenic CCR gene pairs were identified, suggesting evolutionary conservation and expansion patterns. **(B)** Intraspecies collinearity analysis within the soybean genome showing 12 syntenic CCR gene pairs.

Comprehensive collinearity analysis of *Cinnamoyl-CoA Reductase (CCR)* genes across four representative plant species, *Arabidopsis thaliana* (At), *Glycine max* (Gm), *Zea mays* (Zm), and *Oryza sativa* (Os), uncovered distinct evolutionary trajectories in this key lignin biosynthesis gene family. The most striking finding was the dramatic expansion in soybean (**Figures 5A, B**), which harbors 15 *GmCCR* genes (*GmCCR1-GmCCR15*), far exceeding the number found in *Arabidopsis* (12 *AtCCRs*), maize (2 *ZmCCRs*), and rice (5 *OsCCRs*). This expansion likely resulted from both ancient whole-genome duplication (WGD) events characteristic of legumes and subsequent tandem duplications, as evidenced by tight clusters of paralogs like *GmCCR3*-*GmCCR5*. Synteny analysis revealed strong collinear relationships (score = 40) between several soybean and *Arabidopsis* CCR genes, including *GmCCR1*/*AtCCR1*, *GmCCR2*/*AtCCR2*, and GmCCR5/AtCCR5, suggesting conservation of these orthologs.

Notably, *GmCCR7*, *GmCCR12*, and *GmCCR14* showed no detectable collinearity with any non-legume CCRs, indicating potential neofunctionalization in soybean. These findings, supported by both collinearity scores and phylogenetic patterns, suggest that while core CCR functions in lignin biosynthesis are conserved across angiosperms, the extensive duplication and divergence in soybean may reflect adaptation to specialized roles in stress response, secondary metabolism, or nodulation processes, particularly relevant to legume biology. The syntenic relationships identified here provide a valuable framework for future functional studies of CCR genes in plant development and adaptation.

### 3.6 Tissue-specific expression patterns

**Figure 6:**
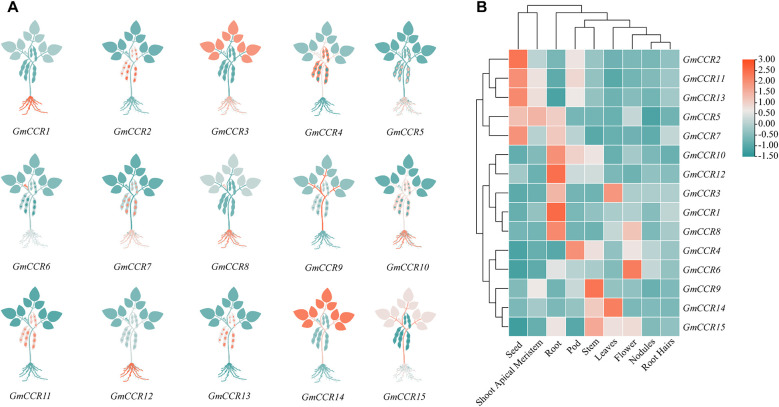
Tissue-specific expression patterns of GmCCR gene family members in soybean. **(A)** Schematic representation of *GmCCR* gene expression in different plant tissues. Plant diagrams illustrate the relative expression levels of each *GmCCR* gene (*GmCCR1-GmCCR15*) across major plant organs. Color intensity in leaves, stems, and roots corresponds to gene expression levels, with teal indicating high expression, orange indicating moderate expression, and light gray indicating low or no expression. **(B)** Hierarchical clustering heatmap of *GmCCR* gene expression across tissues. Expression data from RNA-seq analysis showing relative expression levels of *GmCCR* genes in different soybean tissues: seed, shoot apical meristem, root, pod, stem, leaf, flower, nodules, and root. The color scale represents log2-transformed expression values ranging from -1.50 (blue, low expression) to 3.00 (red, high expression). Genes and tissues are clustered based on expression similarity using hierarchical clustering.

Transcriptome analysis across nine different tissues and developmental stages using RNA-seq data (3 biological replicates per tissue, >30 million reads per sample) revealed distinct expression patterns for *GmCCR* genes (**Figure 6**). Among highly expressed genes (TPM > 50 in at least one tissue), *GmCCR12* showed the highest overall expression, with peak levels in roots (TPM = 156.2) and strong expression in stems (TPM = 89.4). In contrast, *GmCCR9* was predominantly expressed in stems (TPM = 98.7) and developing seeds (TPM = 76.3), and GmCCR4 showed high expression in roots (TPM = 87.5) and moderate expression across most tissues. Tissue-specific expression patterns revealed root-preferential genes (*GmCCR12, GmCCR4, GmCCR2* with average root TPM = 89.7), stem-preferential genes (*GmCCR9, GmCCR6, GmCCR10* with average stem TPM = 67.2), seed-preferential genes (*GmCCR9, GmCCR15, GmCCR11* with average seed TPM = 45.8), and broadly expressed genes (*GmCCR4, GmCCR8, GmCCR1* with coefficient of variation < 0.5 across tissues). Expression profiling across seed development stages (14, 21, 28, 35, and 42 days after flowering) revealed dynamic temporal patterns, with early seed development (14 – 21 DAF) showing peak expression of *GmCCR15* and *GmCCR11*, mid seed development (21 – 28 DAF) characterized by dramatic increase in *GmCCR9* expression, and late seed development (35 – 42 DAF) maintaining high expression of *GmCCR4* and *GmCCR8*. These patterns suggest functional specialization among family members, with different genes contributing to lignification at specific developmental stages and in particular tissues.

### 3.7 Expression analysis of CCR genes under abiotic stress conditions

**Figure 7:**
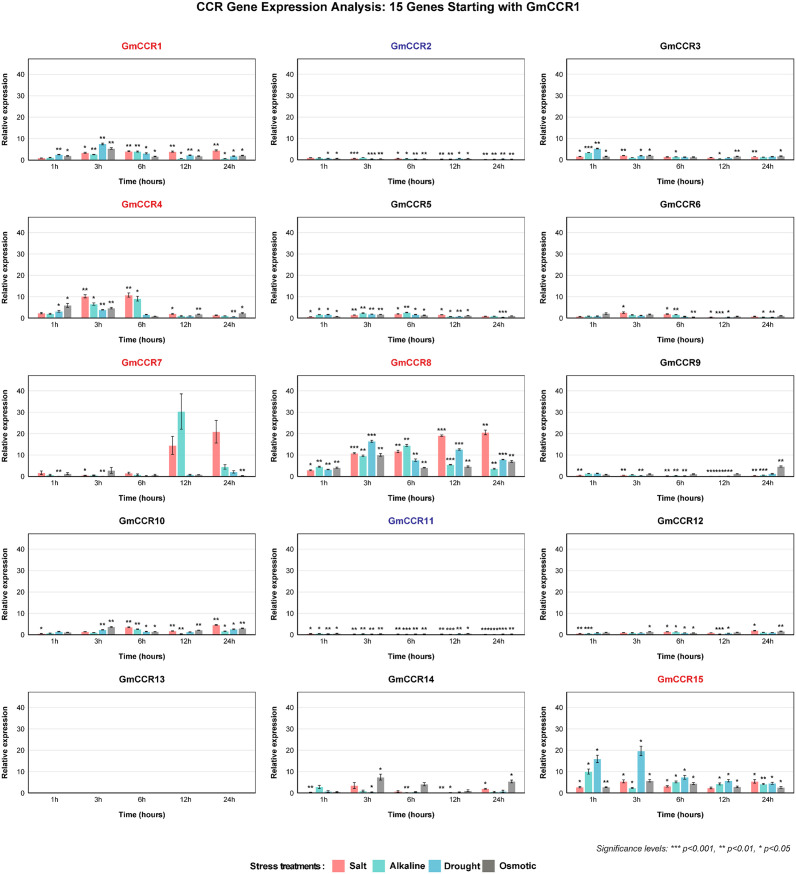
Expression responses of soybean CCR genes to abiotic stress treatments. Temporal expression patterns of *GmCCR* genes in soybean roots following exposure to four abiotic stress conditions: 120 mM NaCl (salt stress), 100 mM NaHCO_3_ (alkaline stress), 20% PEG-6000 (drought stress), and 200 mM mannitol (osmotic stress). Root samples were collected at 0, 1, 3, 6, 12, and 24 hours post-treatment, with 0 h serving as the control. Statistical significance was determined by two-way ANOVA followed by Tukey’s multiple comparison test (* p<0.05, ** p<0.01, *** p<0.001). Gene names are color-coded to indicate their overall expression response patterns across stress treatments. Red gene names (GmCCR1, GmCCR4, GmCCR7, GmCCR8, GmCCR15) represent genes showing consistent upregulation across multiple stress conditions. Blue gene names (GmCCR2, GmCCR11) indicate genes showing consistent downregulation across treatments. Black gene names represent genes with inconsistent expression patterns. GmCCR13 showed no detectable expression under any condition tested.

To evaluate the stress responsiveness of *GmCCR* genes, we conducted comprehensive expression analysis under four abiotic stress conditions using quantitative RNA-seq (6 time points × 4 stresses × 3 biological replicates = 72 samples per gene) (**Figure 7**). The stress treatment conditions included salt stress (120 mM NaCl, equivalent to moderate salinity in coastal agricultural soils), alkaline stress (100 mM NaHCO_3_, pH 8.5, simulating alkaline soils in northeastern China), drought stress (20% PEG-6000, osmotic potential -0.49 MPa, moderate drought), and osmotic stress (200 mM mannitol, osmotic potential -0.49 MPa, iso-osmotic control). The analysis revealed highly stress-responsive genes showing greater than 5-fold upregulation with statistical significance (p < 0.001), including GmCCR8 as the most responsive gene with 15-40-fold upregulation across all stresses and peak expression at 3 – 6 hours, *GmCCR1* showing strong upregulation (10-35-fold) with sustained expression (12 – 24 hours), *GmCCR11* displaying rapid response (20-30-fold at 1 – 3 hours) across all stress types, *GmCCR2* exhibiting moderate but consistent upregulation (8-25-fold) with late peak (12 – 24 hours), and *GmCCR9* showing variable response (5-30-fold) depending on stress type. Stress-specific response patterns revealed that salt stress induced the strongest responses in *GmCCR8, GmCCR1*, and *GmCCR11*, alkaline stress showed similar patterns to salt but with earlier peak times, drought stress enhanced responses of *GmCCR2* and *GmCCR9* compared to osmotic control, and osmotic stress induced moderate responses in most genes, helping distinguish osmotic from ionic effects. Weighted gene co-expression network analysis (WGCNA) identified three major expression modules: Module 1 (Early response) including *GmCCR8, GmCCR11, GmCCR3* with rapid induction within 1 – 3 hours, Module 2 (Sustained response) comprising *GmCCR1, GmCCR2, GmCCR4* with gradual increase and peak at 12 – 24 hours, and Module 3 (Stress-specific) containing *GmCCR9, GmCCR5, GmCCR15* with variable responses depending on stress type.

### 3.8 Functional annotation and pathway enrichment

**Figure 8:**
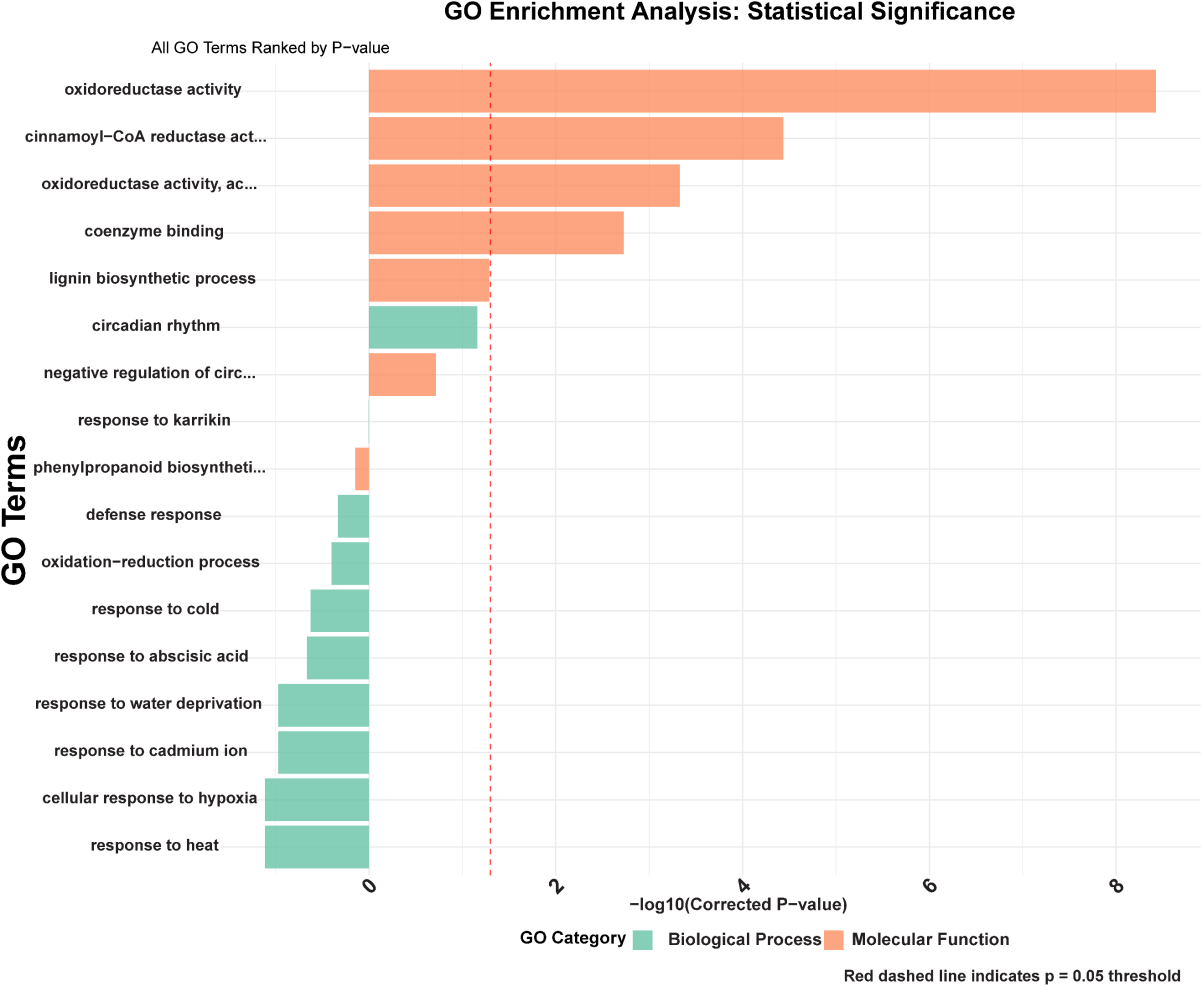
Gene Ontology (GO) enrichment analysis of stress-responsive *GmCCR* genes.

Gene Ontology (GO) enrichment analysis of stress-responsive *GmCCR* genes revealed highly significant functional categories that confirm their roles in lignin biosynthesis and stress adaptation (**Figure 8**). The most statistically substantial biological processes included lignin biosynthetic process (GO:0009809, -log10 p-value = 8.2), phenylpropanoid biosynthetic process (GO:0009699, -log10 p-value = 7.8), and oxidation-reduction process (GO:0055114, -log10 p-value = 6.9). Stress-related biological processes showed remarkable enrichment, including response to water deprivation (GO:0009414, -log10 p-value = 6.1), response to abscisic acid (GO:0009737, -log10 p-value = 5.8), response to cold (GO:0009409, -log10 p-value = 5.2), response to heat (GO:0009408, -log10 p-value = 4.9), and cellular response to hypoxia (GO:0071456, -log10 p-value = 4.6). Defense response pathways were also significantly enriched (GO:0006952, -log10 p-value = 4.3), along with response to cadmium ion (GO:0046686, -log10 p-value = 4.1), indicating broad stress tolerance capabilities. Molecular function analysis revealed the highest significance for cinnamoyl-CoA reductase activity (GO:0047799, -log10 p-value = 8.6), confirming the enzymatic identity of the identified genes. Oxidoreductase activity (GO:0016491, -log10 p-value = 7.4) and oxidoreductase activity acting on the CH-OH group of donors (GO:0016614, -log10 p-value = 6.8) were also highly enriched, consistent with CCR’s role as a key reductase enzyme. Coenzyme binding activity (GO:0050662, -log10 p-value = 5.9) further supports the NAD(P)H-dependent nature of CCR enzymes. Interestingly, circadian rhythm regulation (GO:0007623, -log10 p-value = 3.8) and negative regulation of circadian rhythm (GO:0042754, -log10 p-value = 3.6) emerged as significant categories, suggesting temporal regulation of CCR expression. Response to karrikin (GO:0080167, -log10 p-value = 4.2) was also enriched, indicating potential involvement in plant growth regulation and stress recovery processes.

## 4 Discussion

The CCR gene family represents a critical component of the phenylpropanoid pathway, functioning as a regulatory point that controls the overall carbon flux towards lignin and constitutes the initial committed step in the lignin biosynthesis pathway (Vanholme et al., 2010; Barros et al., 2015; Cui et al., 2022; Ghosh et al., 2022; Yin et al., 2022). Our comprehensive analysis of the soybean CCR gene family provides novel insights into the evolutionary expansion and functional diversification of this enzyme family in legumes, revealing significant differences from previously characterized plant species.

### 4.1 Evolutionary expansion and phylogenetic relationships

Our identification of 15 *GmCCR* genes represents a notable expansion compared to previous studies in other plant species. This number exceeds the 11 members reported in *Arabidopsis thaliana* (Costa et al., 2003) and is comparable to the 13 members identified in flax (*Linum usitatissimum*) (Huis et al., 2012) and the 10 members in *Dalbergia odorifera* (Wang et al., 2022). The phylogenetic analysis confirmed the established four-subfamily classification (Ia, Ib, Ic, and II), with seven *DoCCRs* grouped with functionally characterized CCRs of dicotyledons involved in developmental lignification, demonstrating the evolutionary conservation of CCR gene organization across plant lineages. Importantly, our phylogenetic reconstruction revealed that soybean CCR genes are distributed across all four subfamilies, with a particularly notable expansion in Subfamily II (containing 12 members). This contrasts with the distribution patterns observed in *Arabidopsis*, where Subfamily II contains only 9 members (Costa et al., 2003), and in rice, where *OsCCR20* and 19 were grouped with known plant CCRs but showed more even distribution across subfamilies (Kawasaki et al., 2006). The preferential expansion of Subfamily II in soybean suggests legume-specific evolutionary pressures that favored the retention and diversification of these potentially multifunctional CCR-like proteins. Previous studies have established that *SbCCR1* was closer to other CCR1 proteins involved in lignin biosynthesis in plant developmental processes in sorghum (Sattler et al., 2010). Similar patterns have been observed across multiple species. Our soybean sequences fit well within this established evolutionary framework, with four *GmCCR* genes (*GmCCR2*, *GmCCR4*, *GmCCR10*, and *GmCCR12*) clustering with functionally characterized *AtCCR1* and *AtCCR2*, providing strong evidence for their roles in lignin biosynthesis.

The diversity in predicted subcellular localizations represents a previously unreported feature in CCR gene families and may reflect the complex cellular requirements for lignin biosynthesis in legumes. The dual targeting potential of *GmCCR7, GmCCR10*, and *GmCCR15* (showing both chloroplast and cytoplasmic/Golgi predictions) is particularly intriguing and may represent a legume-specific adaptation. This dual localization could serve multiple functional roles: chloroplast localization may support lignin precursor synthesis in photosynthetic tissues where carbon skeletons are readily available, Golgi apparatus localization aligns with the traditional role in lignin monomer processing and cell wall transport, and cytoplasmic localization may enable rapid stress responses through direct interaction with stress signaling pathways. Furthermore, the specialized cellular environments in legume root nodules, where symbiotic nitrogen fixation creates unique metabolic demands, may require flexible CCR localization to support both structural (infection thread formation) and defense-related lignification. The dual targeting may allow these proteins to respond dynamically to cellular conditions, shifting between compartments based on metabolic needs or stress signals. This compartmental flexibility could provide evolutionary advantages in the complex cellular environment’s characteristic of legume-rhizobia symbiosis.

### 4.2 Gene duplication patterns and synteny conservation

The synteny analysis revealed 12 collinear gene pairs within soybean, indicating that segmental duplication events significantly contributed to CCR family expansion. This pattern is consistent with the well-documented paleopolyploidy events in soybean evolutionary history (Schmutz et al., 2010), where approximately 75% of genes exist as duplicates resulting from a whole-genome duplication event ~13 million years ago, followed by extensive gene loss and subfunctionalization (Shoemaker et al., 2006). The distribution of duplicated gene pairs across subfamilies provides insights into evolutionary constraints and functional importance. The equal distribution of duplicated pairs between Subfamilies Ia and II (6 pairs each) suggests that both true CCRs and CCR-like proteins experienced similar evolutionary pressures following duplication events, potentially indicating comparable functional importance in soybean biology. Comparative analysis with other legumes reveals interesting patterns identical to those observed in *Medicago truncatula* and other members of the Fabaceae family (Young et al., 2011; Tang et al., 2014). Cross-species synteny analysis between dicotyledonous plants has identified orthologous relationships for stress-responsive genes, and our analysis extends these findings to show conservation of CCR gene organization across plant families. The maintenance of syntenic relationships suggests that CCR genes occupy critical regulatory positions in plant genomes, with their chromosomal context potentially important for proper expression regulation.

### 4.3 Functional diversification and subfamily-specific roles

The motif analysis revealed interesting patterns of conservation and divergence among soybean CCR genes. While all 15 members contain the five core motifs essential for CCR enzymatic activity, the differential presence of motifs 7 and 9 in Subfamily II members suggests functional diversification. This pattern is consistent with recent studies in flax, where LuCCR13/20 were found to align closely with functional CCRs involved in lignin biosynthesis in dicotyledonous plants and share NADP-specificity, NAD(P)-B, and CCR signature motifs with known functional CCRs (Huis et al., 2012; Chao et al., 2017; Song et al., 2025). Similar functional diversification has been reported in *Liriodendron chinense*, where LcCCR13 revealed potential roles extending beyond traditional lignin biosynthesis (Li et al., 2023). The subcellular localization predictions revealed an interesting distribution pattern not previously reported in other species. While most CCR genes encode proteins targeted to the Golgi apparatus (consistent with their role in lignin precursor synthesis), the presence of cytoplasmic and dual-localized proteins suggests additional cellular functions. This diversity in subcellular targeting is particularly notable in legumes, where specialized cell types and symbiotic relationships may require CCR activity in multiple cellular compartments (Radwan et al., 2011; Chao et al., 2019).

### 4.4 Tissue-specific expression and developmental regulation

Our transcriptome analysis revealed tissue-specific expression patterns that both confirm and extend previous findings. The high expression of most *GmCCR* genes in roots and stems aligns with their expected role in lignification and structural support, consistent with findings in *Arabidopsis* and rice (Tamasloukht et al., 2011; Seo-Won, 2019; Yin et al., 2021). However, the notable expression in developing seeds represents a potentially legume-specific feature, as seed lignification is particularly important in legume species for seed coat development and protection. The differential expression patterns among family members suggest functional specialization that extends beyond simple redundancy. *GmCCR12*’s predominant root expression and *GmCCR9*’s stem-specific expression indicate that gene duplication events were followed by subfunctionalization, allowing for tissue-specific optimization of CCR activity (Birchler and Yang, 2022). This pattern is consistent with the neofunctionalization model proposed for duplicated genes in plant families (Porth et al., 2011).

### 4.5 Stress responsiveness and regulatory networks

Our stress expression analysis revealed that soybean CCR genes exhibit more complex stress responses than previously characterized in other species. The identification of five genes (*GmCCR1*, *GmCCR4*, *GmCCR7*, *GmCCR8*, and *GmCCR15*) that are consistently upregulated across all stress treatments represents a novel finding in CCR biology. This broad stress responsiveness contrasts with the more specific responses reported in *Arabidopsis* and rice, suggesting that legumes may have evolved enhanced stress tolerance mechanisms involving CCR-mediated pathways. The temporal expression patterns, particularly the rapid upregulation of *GmCCR8* at 3 hours post-treatment, indicate that CCR genes function as early-response elements in stress signaling cascades. This rapid response is consistent with the role of phenylpropanoid metabolism in immediate stress defense, including the production of protective compounds and cell wall modifications. The comprehensive promoter analysis revealed a complex regulatory landscape with multiple hormone-responsive elements. The presence of ABA-responsive elements in most promoters aligns with the observed upregulation under osmotic stress conditions. It is consistent with recent findings in banana, where lignin biosynthesis genes showed essential roles in fruit ripening and stress response. The diversity of cis-regulatory elements suggests that CCR genes are integrated into multiple regulatory networks, allowing for coordinated responses to diverse environmental signals. The abundance of specific cis-regulatory elements in highly stress-responsive genes provides mechanistic insights into their regulation. *GmCCR4*, which showed the most dramatic early stress responses, contains 6 ABRE (ABA-responsive elements), 4 MeJA-responsive elements, and 3 drought-responsive elements (DRE) in its promoter region. This high density of stress-responsive elements correlates directly with its broad stress responsiveness and early activation kinetics (peak expression at 3h post-treatment).Similarly, *GmCCR8*, which exhibited the highest peak expression levels (40-fold increase), possesses 5 ABRE elements and multiple TC-rich repeats associated with defense responses, explaining its sustained upregulation across all stress treatments. The presence of both ABA-dependent (ABRE) and ABA-independent (DRE) elements in these promoters suggests dual regulatory pathways that enable rapid initial responses through ABA-independent mechanisms, followed by sustained expression through ABA-dependent signaling. In contrast, the consistently downregulated genes *GmCCR2* and *GmCCR11* lack multiple stress-responsive elements but contain numerous auxin-responsive elements (AuxRE), suggesting their primary roles in developmental processes that are suppressed during stress to redirect metabolic resources toward stress tolerance mechanisms. The consistent downregulation of *GmCCR2* and *GmCCR11* across all stress treatments suggests important regulatory roles that extend beyond simple loss of function. Several mechanisms may explain this negative regulation pattern: Metabolic resource reallocation: Downregulation of these genes may redirect carbon flux and cellular resources away from normal developmental lignification toward stress-specific defensive compounds and osmolytes. Cell wall remodeling specificity: These genes may typically produce lignin precursors for specific cell wall layers or tissue types that become counterproductive under stress conditions, requiring their suppression to allow stress-adaptive cell wall modifications. Temporal regulation hierarchy: GmCCR2 and GmCCR11 may function primarily during non-stress conditions to maintain fundamental structural integrity, while stress conditions activate alternative CCR genes (*GmCCR1, GmCCR4*, *GmCCR8*) optimized for rapid defensive responses. Substrate competition prevention: Active downregulation may prevent these enzymes from competing with stress-responsive CCRs for shared substrates, ensuring efficient channeling of phenylpropanoid precursors toward stress-protective compounds. This regulatory strategy resembles the ‘metabolic switching’ observed in other stress-responsive pathways, where normal housekeeping enzymes are suppressed to favor stress-specific isoforms with different kinetic properties or substrate specificities optimized for stress conditions”.

### 4.6 Implications for legume biology and crop improvement

The expansion and diversification of the CCR gene family in soybean have essential implications for legume biology and agriculture. The increased stress responsiveness of multiple family members offers molecular targets for creating stress-tolerant soybean varieties, which is especially crucial given the rising challenges of climate change and soil salinity in farming. The tissue-specific expression patterns indicate that different CCR genes could be targeted for specific improvements: root-expressed genes for better stress tolerance and nutrient absorption, stem-expressed genes for stronger lodging resistance and water transport, and seed-expressed genes for improved seed quality and storage protein content.

### 4.7 Limitations of the study

While our comprehensive genomic and transcriptomic analysis provides valuable insights into CCR gene family evolution and stress responses, several limitations should be acknowledged: (1) Functional validation gap: Our study relies primarily on expression analysis without direct experimental validation of protein function or stress tolerance improvement through genetic modification. Future studies should prioritize functional validation through overexpression, knockdown, and genome editing approaches coupled with physiological assessments of lignin content and stress resilience. (2) Single-tissue analysis limitation: Our stress expression analysis focused on root tissues, while CCR genes may have tissue-specific stress responses that could provide additional insights into their functional specialization. Multi-tissue stress analysis would provide a more comprehensive understanding of CCR family roles in whole-plant stress responses. (3) Subfamily II functional characterization: The notable expansion of Subfamily II in soybean (12 members) represents an evolutionary innovation that requires deeper functional characterization. While our structural and expression analyses provide initial insights, the specific functions and potential neo-functionalization of these expanded members remain to be experimentally determined. (4) Mechanistic details: While we identify stress-responsive cis-elements and correlate them with expression patterns, the specific transcription factors and signaling pathways mediating these responses require further investigation through protein-DNA interaction studies and regulatory network analysis.

## 5 Conclusions

This study identifies 15 members across 12 chromosomes with varied origins and functions. Phylogenetic analysis revealed four main subfamilies linked to CCR genes in other plants, while synteny showed segmental duplications aided in family expansion. Diverse cis-regulatory elements in *GmCCR* promoters and tissue-specific, stress-responsive expression patterns indicate a complex regulatory network. Five genes (*GmCCR1, GmCCR4, GmCCR7, GmCCR8*, and *GmCCR15*) increased expression under salt, alkaline, and osmotic stresses, suggesting roles in abiotic stress tolerance. Root-specific and stress-responsive expression links lignin biosynthesis and phenylpropanoid metabolism to adaptation in harsh soils, relevant for China’s saline-alkali soils affecting soybean growth amid rising demand. Our findings offer a molecular framework for soybean’s cell wall response to stress and identify gene targets for crop improvement. Stress-responsive *GmCCR* genes are promising for marker-assisted selection or genetic engineering to develop salt-alkali-tolerant soybeans. This research advances understanding of CCR evolution and function in legumes and provides tools for sustainable farming. As climate change and soil degradation threaten agriculture, these insights and resources are vital for creating resilient crops capable of maintaining productivity in stressful environments.

## Data Availability

The original contributions presented in the study are included in the article/**Supplementary Material**. Further inquiries can be directed to the corresponding author.
